# Primary cilia: Versatile regulator in cartilage development

**DOI:** 10.1111/cpr.12765

**Published:** 2020-02-08

**Authors:** Fenghua Tao, Ting Jiang, Hai Tao, Hui Cao, Wei Xiang

**Affiliations:** ^1^ Department of Orthopedics Renmin Hospital of Wuhan University Wuhan University Wuhan China; ^2^ Department of Neurological Rehabilitation Zhongnan Hospital of Wuhan University Wuhan University Wuhan China

**Keywords:** cartilage development, cartilage tumorigenesis, mechanical stress, osteoarthritis, primary cilia, signalling transduction

## Abstract

Cartilage is a connective tissue in the skeletal system and has limited regeneration ability and unique biomechanical reactivity. The growth and development of cartilage can be affected by different physical, chemical and biological factors, such as mechanical stress, inflammation, osmotic pressure, hypoxia and signalling transduction. Primary cilia are multifunctional sensory organelles that regulate diverse signalling transduction and cell activities. They are crucial for the regulation of cartilage development and act in a variety of ways, such as react to mechanical stress, mediate signalling transduction, regulate cartilage‐related diseases progression and affect cartilage tumorigenesis. Therefore, research on primary cilia‐mediated cartilage growth and development is currently extremely popular. This review outlines the role of primary cilia in cartilage development in recent years and elaborates on the potential regulatory mechanisms from different aspects.

## 1 INTRODUCTION

Cartilage, an integral part of the skeletal system, has unique functions of secreting the cartilage matrix, buffering stress concussion, conducting weight‐bearing and maintaining the normal activity of joints. Chondrocytes, the main cells in cartilage tissue, are highly differentiated with a limited capacity for proliferation and redifferentiation. It is difficult for chondrocytes to perform self‐repair quickly and effectively after injury or degeneration.^1^ Therefore, promoting cartilage regeneration and injury reparation are urgent and critical challenges in orthopaedic clinical practice. The regulatory mechanism of cartilage development is complex and can be affected by many factors, such as mechanical stress, inflammation, osmotic pressure, hypoxia and signalling transduction.^2^, ^3^, ^4^, ^5^, ^6^ Chondrocytes have biomechanical reactivity and stress tolerance capacities. Mechanical stress is an indispensable stimulus for maintaining the biomechanical properties of cartilage and affecting chondrocyte activities. For example, mechanical stress is crucial in regulating cartilage nutrient acquisition and wastes removal,^7^, ^8^ helping maintain the phenotype and cartilage function,^9^, ^10^, ^11^ and affecting cartilage and limb formation during embryonic development.^12^ Similarly, the occurrence of cartilage‐related diseases and cartilage tumorigenesis can also be affected by complex regulatory mechanisms.

Currently, many studies have linked the development of cartilage with the function of primary cilia. In this review, we present comprehensive views on the roles of primary cilia in cartilage development, especially highlighting the roles of primary cilia in regulating cartilage matrix secretion, endochondral ossification, mechanical signalling transduction, cartilage tumorigenesis and cartilage disease, such as osteoarthritis. An overview regarding the structure and physiological functions of primary cilia precedes the discussion of ciliary‐mediated cartilage development. Detecting the regulatory mechanism of cartilage development and illuminating the pathogenesis of cartilage‐related tumorigenesis and diseases are valuable research topics and may eventually providing new ideas for the treatment of cartilage disease and cartilage injury repair.

## 2 PRIMARY CILIA

Primary cilia, as antenna‐like highly conserved sensory organelles, were once considered incompletely degraded organelles in evolution. They mainly assembly and protrude on the surface of most eukaryotic cell membranes when they progress into the quiescent phase of the cell cycle.^13^ The abnormal expression of primary cilia has been associated with some hereditary diseases, such as polycystic kidney disease, Joubert syndrome, Bardet‐Biedl syndrome, Jeune asphyxiating thoracic dystrophy and short rib polydactyly syndrome.^14^, ^15^, ^16^, ^17^, ^18^ Furthermore, the dysfunction of primary cilia and ciliogenesis disorder is involved in the regulation of diverse tumorigenesis.^19^ In malignant cartilage tumours, aberrant primary cilia are associated with the abnormal proliferation and differentiation of chondrocytes during the process of chondosarcomagenesis.^20^ In benign cartilage tumours, primary cilia are randomly located on osteochondroma cell surfaces, which implies the loss of polarity in this benign tumour.^21^

Moreover, primary cilia, as sensory organelles, have multiple functions, including receiving physical and chemical signals and regulating signalling transduction.^14^, ^17^ Recently, detecting the regulatory mechanism of primary cilia on cartilage development has become a popular topic in the research of cartilage disease and regeneration.^22^, ^23^, ^24^ Exploring the regulatory mechanism of primary cilia is crucial for understanding the pathogenesis of cartilage diseases and can provide new ideas for facilitating cartilage regeneration via regulating ciliogenesis. The purpose of this review is to discuss and summarize the recent findings of primary cilia and their regulatory mechanism on cartilage growth and development.

### 2.1 The structure of primary cilia

**Figure 1:**
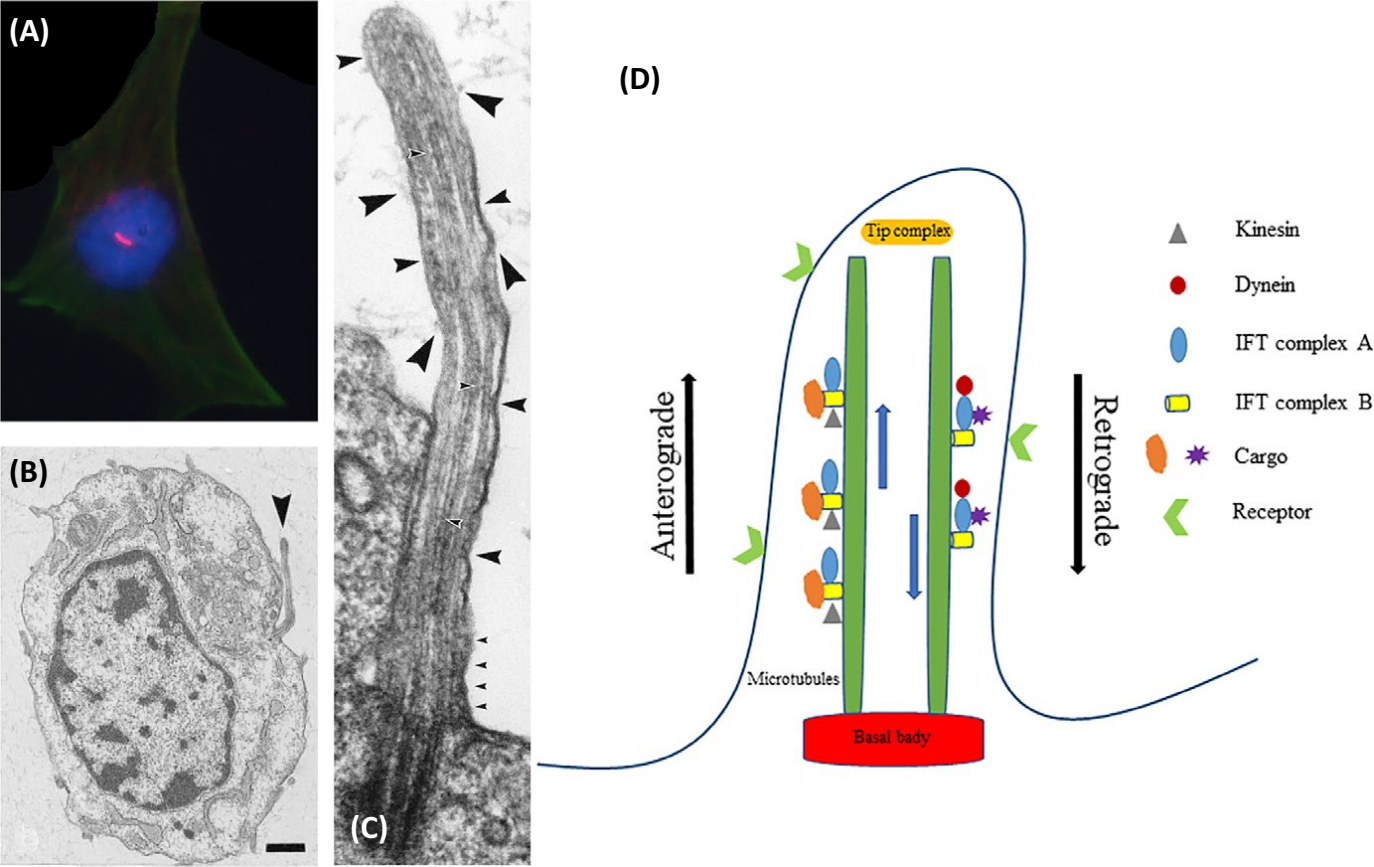
The structure of primary cilia under immunofluorescence staining and electron microscope observation. A, Immunofluorescence staining of primary cilia (red) and F‐actin (green). B, Primary cilia on the cell surface (black arrow). C, Microtubules extending from the basal body to the distal tip of primary cilia and ciliary functional proteins scattered around the adjacent microtubules (black arrows). D, Substance transportation in primary cilia. Kinesin and IFT complex B mediate the anterograde transportation from the cytoplasm to the tip of the primary cilia, and dynein and IFT complex A mediate the retrograde transportation from the tip to the cytoplasm. B and C, were reprinted with permission from Ref.^30^ Copyright 2002 John Wiley and Sons

Primary cilia are antenna‐like structured organelle protruding from cell membranes (Figure 1A,B), which can be divided into two parts: the axon portion and the basal body. The axon portion of the primary cilia protrudes from the surface of the membrane and the basal body anchors to the inner cell membrane (Figure 1C). The skeleton of primary cilia is mainly assembled by "9+0" pairs of microtubules. Primary cilia (one per cell) differ from motile cilia (multiple per cell) in that they lack a central pair of microtubules and the inner and outer dynein arms, and are known as the non‐motile cilia in cell's membrane.^25^, ^26^ Primary cilia are microtubule‐based organelles originating from the basal body, which is a modified form of the centriole. The microtubules of primary cilia are homologous to mitotic spindle filaments that are regulated and organized by the centriole.^27^ Primary cilia protrude in quiescent or differentiated cells and can be treated as indicators for cells in the quiescent phase of mitosis.^28^, ^29^ Furthermore, the microtubules of primary cilia experience acetylated modification during polymerization and assembly and can be observed by the fluorescence staining of acetylated α‐tubulin (Figure 1A).^30^

### 2.2 Substance transportation in primary cilia

In primary cilia, the microtubule assembly, axon elongation and signalling transduction depend on the intraflagellar transport system. Ciliary intraflagellar transport (IFT) proteins dominate the transportation of ciliary substances. This pattern of transport is reciprocated along the axonal microtubules with the help of IFT proteins. As Figure 1D shows, The IFT complex A is transported from the apical apex to the basal body, which is driven by the protein dynein, while the IFT complex B mediates the protein from the basal to the apical forward transportation and kinesin involved in this driving process.^31^ Furthermore, some studies have revealed that the mutation of the core part of IFT complex B can lead to primary cilia disassembly and disturb their biological functions.^31^, ^32^, ^33^ Therefore, the transport function of primary cilia is essential for maintaining the normal function of cells.

### 2.3 The sensory functions of primary cilia

As sensory organelles, many kinds of ion channels and signalling receptors are localized in primary cilia. The biological and physicochemical stimuli in the microenvironment can promote the protein association or dissociation from primary cilia to conduct signalling transduction, which eventually triggers cell activities, such as proliferation and differentiation.^27^ A pressure‐gated ion channelTRPV4 (transient receptor potential vanilloid 4) is anchored to the surface of the primary cilia, which can sense the osmotic pressure in the microenvironment and cause the Ca^2+^ flow to change in and out of articular chondrocytes.^34^ When culturing chondrocytes in a hypertonic or hypotonic environment, primary cilia quickly sink into the basal body to shorten the length of the cilia. However, the responses of chondrocytes to the changes of osmotic pressure can be attenuated by inhibiting TRPV4 activity in primary cilia of articular cartilage.^34^, ^35^ Furthermore, the changes in inflammatory factors in extracellular microenvironments can also be detected by primary cilia. When treating chondrocytes with IL‐1, nitric oxide and prostaglandin E2 releasing as well as primary cilia elongation can be detected in the articular chondrocytes. However, the structure of primary cilia was disrupted, and the inflammatory response of IL‐1 was attenuated significantly in IFT88 mutant cells, indicating that primary cilia were involved in the regulatory process of intracellular inflammatory responses.^36^ Recently, the roles of primary cilia in mechanical signalling transduction have attracted increasing attention. A recent study reported that fluid stress in microenvironments can force primary cilia bending, thereby triggering the opening of calcium sensitive channels to allow Ca^2+^ to flow into the primary cilia. Furthermore, intracellular signalling transduction can be activated by the cascade effect of Ca^2+^ influx, which can affect the downstream gene expression and initiate adaptive changes in cell activities.^37^ In addition, cyclic compressive stress can induce the prevalence and length changes of primary cilia in articular cartilage and facilitate chondrocytes function changes simultaneously.^38^ The sensory functions of primary cilia are indispensable for cells to respond to the physicochemical stimulation in microenvironments to regulate cell growth and development.

### 2.4 Signalling transduction in primary cilia

Recently, nearly 1000 cilia‐related proteins have been screened and identified in primary cilia. Some of these proteins are anchored on the ciliary membrane, some are distributed in the basal body, and some can be transported bi‐directionally along the axons by the IFT complex. Most of these proteins are involved in different signalling pathways with diverse functions.^39^, ^40^ Primary cilia participate in Hedgehog, Wnt, PDGFR, Notch, TGF‐β, mTOR and other signalling transduction.^37^ For example, primary cilia can be treated as an adjustment switch to regulate non‐canonical Wnt signalling transduction. In the non‐canonical Wnt signalling pathway, an external stimulus can act on the primary cilia to increase Ca^2+^ influx into cells, and the ciliary‐related protein inversin distributes to the basal body, which can promote the ubiquitination and degradation of APC/C after targeted binding to cytoplasmic disheveled.^41^, ^42^, ^43^ Platelet‐derived growth factor receptor (PDGFR α), as a G protein‐coupled receptor, is located on the membrane of primary cilia. The PDGFR pathway can be activated through PDGF ligands binding to the receptors and inducing cellular responses through activating downstream MEK/ERK cascades.^44^ In the Notch pathway, the Notch3 receptor is anchored to the primary cilia membrane and can be activated via interacting with presenilin‐2 which is localized at the basal body of primary cilia.^45^ Furthermore, the activation of the Notch pathway can play an important role in regulating primary cilia elongation and affecting structural symmetry.^46^, ^47^ In the presence of TGF‐β ligands, the activated TGF‐β receptors can migrate from the surface of primary cilia to the vicinity of the basal body to activate smad2/3 and up regulate the target gene expression by binding to SMAD4.^48^ The cilia‐dependent TGF‐β pathway can also exert promotive effects by activating ERK1/2 signalling or affecting IFT88 expression in chondrocytes.^49^, ^50^ In addition, the mTOR pathway and primary cilia are involved in the pathogenesis of cilia‐related polycystic kidney diseases.^51^ When primary cilia underwent bending deformation under hydrodynamics condition, the downstream signalling mTORC1 could be blocked through regulating the LKB‐1‐AMPK‐mTOR cascade reaction at the basal body of primary cilia, thereby affecting the volume of cells.^52^ Therefore, it is believed that maintaining the integrity of primary cilia signalling transduction can help regulate the hypertrophy and proliferation of cells and affect the progression of disease.^53^

Additionally, primary cilia have recently been found to have secretory functions. A previous study reported that the exfoliation of photoreceptors was observed, and the phagocytosis of retinal pigment epithelium on the exfoliated disc was confirmed, suggesting that it is possible to exfoliate the membrane or membranous vesicle from the tip of primary cilia.^54^ Later, the budding and release of apical membranous vesicle were found in Chlamydomonas cilia, and the exfoliated ciliary bodies were found in the extracellular microenvironment.^55^ When phosphatidylinositol 5 phosphatase E (INPP5E) was inhibited, the accumulation of phosphatidylinositol 4,5 diphosphate (PI (4,5) P2) in primary cilia led to the formation and shedding of vesicular at the top of the cilia and resulted in the depolymerization of primary cilia.^56^ The extracellular bodies secreted by primary cilia were gradually recognized, but there are still many problems needing to be solved in the near future.

## 3 PRIMARY CILIA IN SKELETAL SYSTEM DEVELOPMENT

Primary cilia regulate the chondrogenesis and osteogenesis processes of skeletal system development in the embryonic stage.^31^, ^57^ They also affect the development of the growth plate cartilage and articular cartilage after birth.^58^, ^59^ During cartilage development, primary cilia are treated as a signalling nexus and considered to be a type of attractive therapeutic target for the diseases or disorders of the skeletal system.^60^ Abnormal disturbance of primary cilia can lead to a series of diseases called "ciliary‐related cartilage dysplasia," such as short rib polydactyly syndrome, which is a kind of hereditary disease characterized by symptoms of dysplasia and mainly affects the development of long bones, ribs and craniofacial bones.^61^, ^62^ Similar phenomena can be found in cilia‐related genes mutants. For example, Tg737 is an important gene that can regulate primary cilia assembly, while the abnormal mutation of Tg737 can cause the loss of cell polarity and lead to primary cilia disassembly. Skeletal developmental disorders have been observed in Tg737 mutated mice. The length and width of the growth plates in mutant mice limbs were shorter than those in wild mice. In addition, the density of articular chondrocytes also changed, accompanied by the abnormal expression of primary cilia as well as cartilage dysplasia.^33^ The knockout of primary cilia functional gene IFT80 can also lead to the loss of primary cilia and cartilage dysplasia. Mice with embryonic knockout of IFT80 had short limbs at birth with decreased chondrocyte volumes and changed chondrocyte morphology in all layers of the growth plate. The knockout of IFT80 after birth can cause cartilage dysplasia characterized by a shortened growth plate and thickened articular cartilage. This phenomenon may be caused by the disturbance of chondrogenic differentiation via inhibiting Hedgehog signalling and activating Wnt signalling.^63^ The dysfunction of the primary cilia can also lead to polydactyly in the Bardet‐Biedl syndrome. The knockout of ciliary proteins Bbs1, Bbs2 and Bbs6 can reduce the thickness of articular cartilage and decrease the secretion of proteoglycan in mutant mice, as well as change the distribution and arrangement of chondrocytes in cartilage tissue.^15^ In addition, the deficiency of kinesin motor complexes Kif3a and Kif5b can cause delayed bone growth and post‐natal dwarfism due to the disorganized columnar structure in the growth plate of the long bone.^59^, ^64^ Therefore, primary cilia play critical roles in maintaining the normal development of the skeletal system.

## 4 PRIMARY CILIA AND CARTILAGE DEVELOPMENT

### 4.1 Primary cilia in cartilage matrix secretion

Cartilage is an indispensable tissue for maintaining the proper functioning of the body skeletal system, such as the resisting of overloaded mechanical stress, helping maintain normal joint activity and promoting longitudinal bone growth. Chondrocytes achieve their physiological functions by synthesizing and secreting a cartilage matrix which predominantly contains of collagen fibres and glycosaminoglycan to enhance tensile strength and hydration.^65^ However, the environment in which cartilage growth occurs lacks vessels and is deficient in oxygen and nutrients. Chondrocytes are highly differentiated cells with limited proliferative ability and easily to dedifferentiate and degenerate under certain conditions. The regulatory mechanism of chondrocyte development and adaptation to the complex microenvironment remains unclear. Primary cilia were once considered organelles that had not degenerated completely but have been confirmed as serving diverse functions recently, such as sensing external stimulus and mediating signalling transduction. The important roles of primary cilia in regulating cartilage growth and development have gradually been recognized.

**Figure 2:**
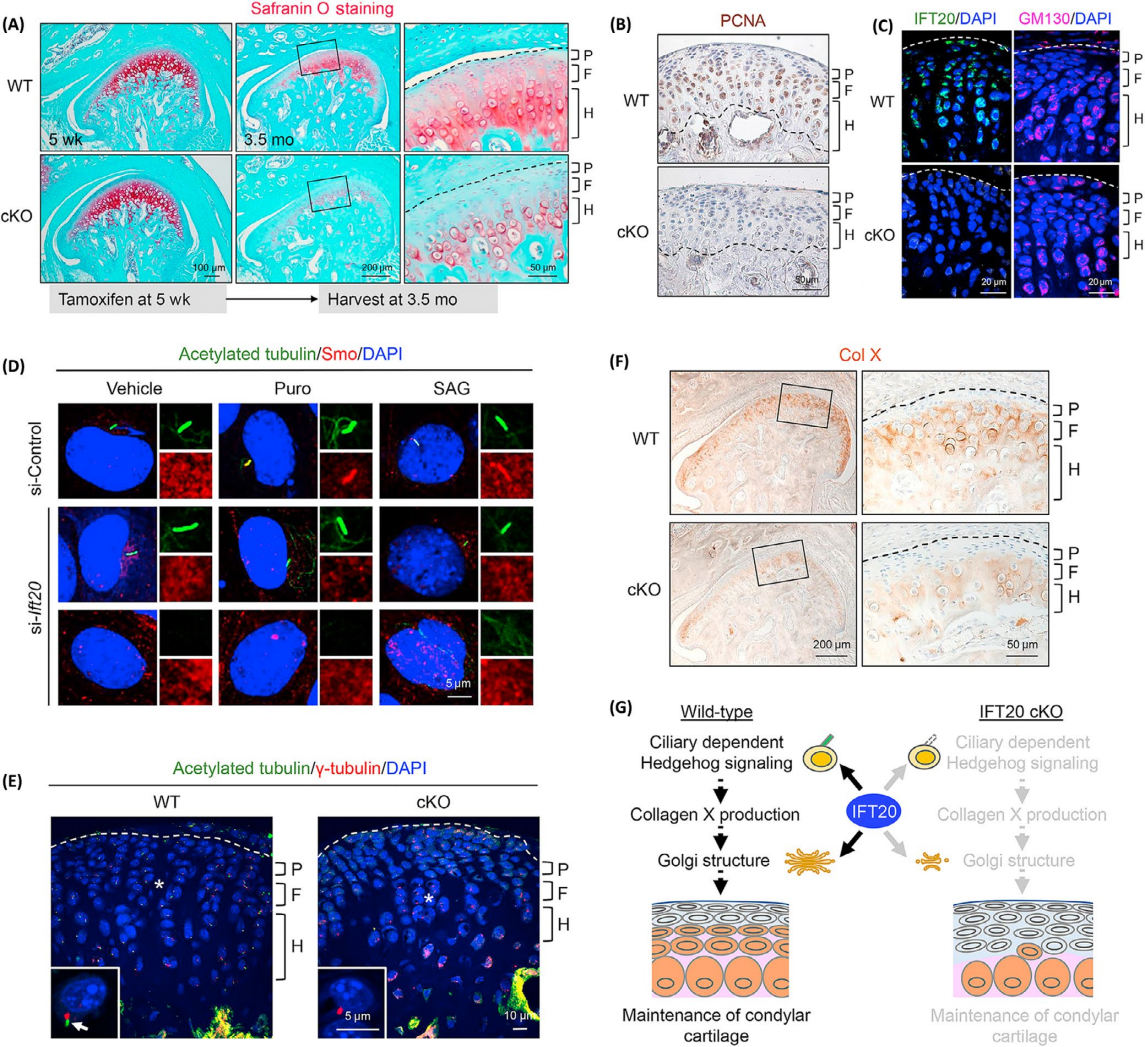
Primary cilia functional protein IFT20 is required for cartilage matrix maintenance in condylar cartilage. A, Safranin O staining in the mandibular condyle of Ift20 conditional knockout mice (cKO) and wild‐type controls (WT). B, PCNA immunohistochemistry in Ift20 cKO and WT mice. C, The localization of IFT20 and the size of GM130 (cis‐Golgi marker) in condylar cartilage. D, Primary cilia and Hedgehog signalling‐related Smo protein expression in ATDC5 cells were transfected with si‐Ift20 and treated with Hedgehog agonist Purmorphamine (Puro) or Smoothened agonist (SAG). E, Primary cilia in the cartilage tissues of WT and Ift20 cKO mice. F, COL‐X expression in WT and Ift20 cKO mice. G, The potential mechanism of IFT20 is to regulate the Golgi size and Hedgehog signalling transduction to control the maintenance of the condylar cartilage. Reprinted with permission from Ref.^67^ Copyright 2019 Elsevier

Primary cilia are involved in the regulation of articular cartilage matrix secretion. Early studies confirmed that the secretion of the cartilage matrix is regulated by the signalling axis of the cartilage matrix‐primary cilia‐Golgi apparatus. Primary cilia can act as a "bridge" in this process and help transmit environmental information from the external matrix to regulate the exocytosis of the Golgi vesicles.^30^ The basal body of the primary cilia was observed physiologically connected to the Golgi apparatus in chondrocytes, which implied that the secretory function of the extracellular matrix could be regulated, in part, by primary cilia.^66^ Furthermore, the ciliary functional intraflagellar transport protein IFT20 is essential in regulating Hedgehog signalling transduction and affecting Golgi size to maintain a cartilaginous matrix and maintain the homeostasis of condylar cartilage (Figure 2).^67^ Disruption of ciliary trafficking protein IFT88 can also affect the extracellular protease activity and regulate the extracellular matrix remodelling of articular chondrocyte via regulating LRP‐1‐mediated endocytosis.^65^

The elongation of longitudinal bone is inseparable from the extracellular matrix secretion and calcification in the multilayered growth plate at the metaphysis. Primary cilia play indispensable roles in the regulation of proliferation, hypertrophy and cartilage matrix secretion in the oriented column‐like chondrocytes of the growth plate. The knocking out of the Tg737 gene can disrupt ciliogenesis. The growth plates in articular epiphyseal of Tg737orpk mice were smaller in width and length, and a marked decreased secretion of collagen X delayed the hypertrophy differentiation of chondrocytes.^33^ The normal function of the IFT/cilia axis can contribute to maintaining the columnar organization of the growth plate. The disruption of IFT inhibits proliferation but enhances aggrecan and collagen X secretion to accelerate the hypertrophic differentiation in growth plates of articular epiphyseal.^59^ Xue et al^63^ also revealed that short limbs could be observed in IFT80‐deficient mouse models with a reduction of proteoglycans secretion and a shortened cartilage in tibias growth plates. Therefore, the primary cilia mediating cartilage matrix secretion and hypertrophic differentiation in growth plates is particularly critical for the development of limbs, especially promoting the normal programme of endochondral ossification.

### 4.2 Primary cilia in endochondral ossification

Endochondral ossification is one of the two essential processes of bone development in vivo. Recently, primary cilia have been found to be important cellular organelles in regulating endochondral ossification. Generally, the mutations of the Pkd1 gene are associated with the occurrence of primary cilia‐related polycystic kidney disease, and mice with Pkd1 mutations can exhibit endochondral ossification disorders in the embryonic stage. Delaying growth plate chondrocyte hypertrophy differentiation and bone mineralization, the shortening of long bones, and vertebral dysplasia can be observed in these Pkd1 mutant mice.^68^ In IFT88 mutant mice, the disorder of embryonic endochondral ossification can also be observed, which has been found accompanied by accelerating hypertrophy differentiation of the growth plate chondrocytes and delayed angiogenesis in the ossification center.^58^, ^69^ The defective expression of ciliary kinesin II motor complex Kif3 cause post‐natal dwarfism because the reduced proliferation and accelerated hypertrophic differentiation of chondrocytes can result in the abnormality of endochondral ossification in the growth plate of long bones. Furthermore, chondrocytes exhibited alterations in cell orientation and shape, as well as the loss of columnar organization within the growth plate of lone bones in Kif3a mutant mice.^59^ Additionally, deficiency of G protein‐coupled receptor Gpr161 on the surface of primary cilia can cause chondrocytes to lose the ability to differentiate into columnar chondrocytes, which can eventually lead to the disorder of endochondral ossification and affect the development of embryonic forelimbs.^70^ The development of long bones is inseparable from endochondral ossification, and it is a complex regulatory process. The detailed mechanisms of primary cilia‐mediated endochondral ossification need to be fully deciphered, especially for elucidating the regulatory mechanism of signalling pathways and other factors.

### 4.3 Primary cilia in cartilage mechanical signalling transduction

Various kinds of mechanical stress exist in the extracellular environment, such as compressive stress, tensile stress, hydrostatic pressure and fluid shear stress.^71^, ^72^, ^73^, ^74^, ^75^ Mechanical stress is an essential physical stimulus in regulating cell growth and development. Cells undergoing mechanical stress need to respond appropriately to avoid damage caused by the changes in the mechanical environment. Primary cilia have been found crucial in biomechanical signalling transduction and are recognized as mechanosensors.^27^ When chondrocytes suffer from mechanical stress, primary cilia bending can be observed, and this change is considered the response of chondrocytes to mechanical stress, which eventually facilitates the secretion of the extracellular matrix.^76^

Furthermore, cartilage matrix can be periodically compressed and relaxed under mechanical stress, causing water content and osmotic pressure changes in the proteoglycan space of the cartilage matrix in a short time. Primary cilia protruding on the surface of chondrocytes can respond to mechanical stress‐induced osmotic pressure changes by changing the length. The results indicate that primary cilia have good biomechanical reactivities under mechanical stress conditions.^35^

#### 4.3.1 Mechanical stress‐sensitive receptors on primary cilia

A variety of mechanoreceptors is embedded in the membranes of primary cilia, which are involved in mediating intracellular and extracellular mechanical signal transduction and ultimately regulate cell growth and development.^34^ TRPV4, an ion channel on primary cilia, can sense mechanical stress‐induced osmotic pressure changes in the cartilage matrix. TRPV4 can then initiate intracellular calcium flow changes so that cells can make timely and appropriate responses to external stimuli. Mutations of the TRPV4 gene can cause skeletal system dysplasia and joint degeneration.^77^ Connexin 43, a mechanically sensitive adenosine triphosphate (ATP) release channel located on primary cilia, can initiate ATP release when undergoing mechanical stress and can act as a second messenger binding to purine receptors P2X and P2Y on articular chondrocytes.^78^ Integrin, a cellular bridge between the intracellular skeleton and extracellular matrix, is involved in the transduction process of mechanical signalling.^79^, ^80^ A study previously reported that integrin strengthens the connection with the extracellular matrix by changing the molecular conformation when cells were subjected to external force. Different conformations of integrin molecules α2, α3 and β1 expressed on the primary cilia of chondrocytes were thought to be the key molecules maintaining the connection with the extracellular matrix.^81^ Ca^2+^ channels are also widely distributed on the surface of primary cilia. Mechanical stress can activate Ca^2+^ signalling in cartilage by acting on primary cilia. Intracellular Ca^2+^ flow changes can be treated as a common manifestation of other signalling transduction in response to external mechanical stress. Bending deformation of the primary cilia can be observed when cells are subjected to mechanical stress in the microenvironment. Mechanical sensitive receptors PC1 and PC2 located on the primary cilia membrane can form a stress receptor complex, and the activation of the PC2 receptor can induce the increase of intracellular Ca^2+^ flow in response to external mechanical stimuli.^14^, ^17^ Primary cilia can transduce mechanical signalling by regulating the ATP‐mediated Ca^2+^ pathway under mechanical stress.^82^ Additionally, the TRP channel (transient receptor potential channel) anchored on the surface of the primary cilia can participate in the regulation of Ca^2+^ signalling, which promotes adaptive cell adaptive responses to various physical and mechanical stimuli.^83^

#### 4.3.2 Primary cilia in mechanical stress‐mediated chondrogenesis

Cartilage tissue is a semi‐rigid connective tissue with unique mechanical reactivity. Recently, several studies have proved that primary cilia can be involved in the regulation of mechanical stress‐mediated cartilage development.^84^ When precursor and hypertrophic chondrocytes were subjected to the appropriate intensity of mechanical stress, the expression of COL‐II, COL‐X and BMP‐2 genes was upregulated, while these corresponding genes were inhibited after the disruption of the primary cilia assembly. In contrast, mechanical stress can only promote COL‐X gene expression in hypertrophic chondrocytes, but the disturbance of the ciliary structure did not affect COL‐X expression. The results indicate that primary cilia can regulate precursor chondrocyte differentiation and affect terminally differentiated hypertrophic chondrocyte development under mechanical stress.^85^ He et al revealed that mechanical stress of appropriate intensity can inhibit metalloproteinases MMP‐1 and MMP‐13 expression, and initiate the activation of CITED2 mediated signalling transduction in primary cilia. Furthermore, intra‐articular injection of IFT88 siRNA can reduce the expression of exercise training induced CITED2. The regulatory mechanism should be that mechanical stress transactivates CITED2 through regulating the primary cilia‐ATP‐purine calcium‐ERK1/2 signalling axis.^86^ In adult bovine articular chondrocytes, cyclic tensile strain (10% strain) can activate Hedgehog signalling transduction and affect ADAMTS‐5 expression in a primary cilia‐dependent manner, whereas high‐intensity mechanical stress can induce acetylase 6‐mediated depolymerization of primary cilia and block both Hedgehog signalling and ADAMTS‐5 expression simultaneously.^87^

**Figure 3:**
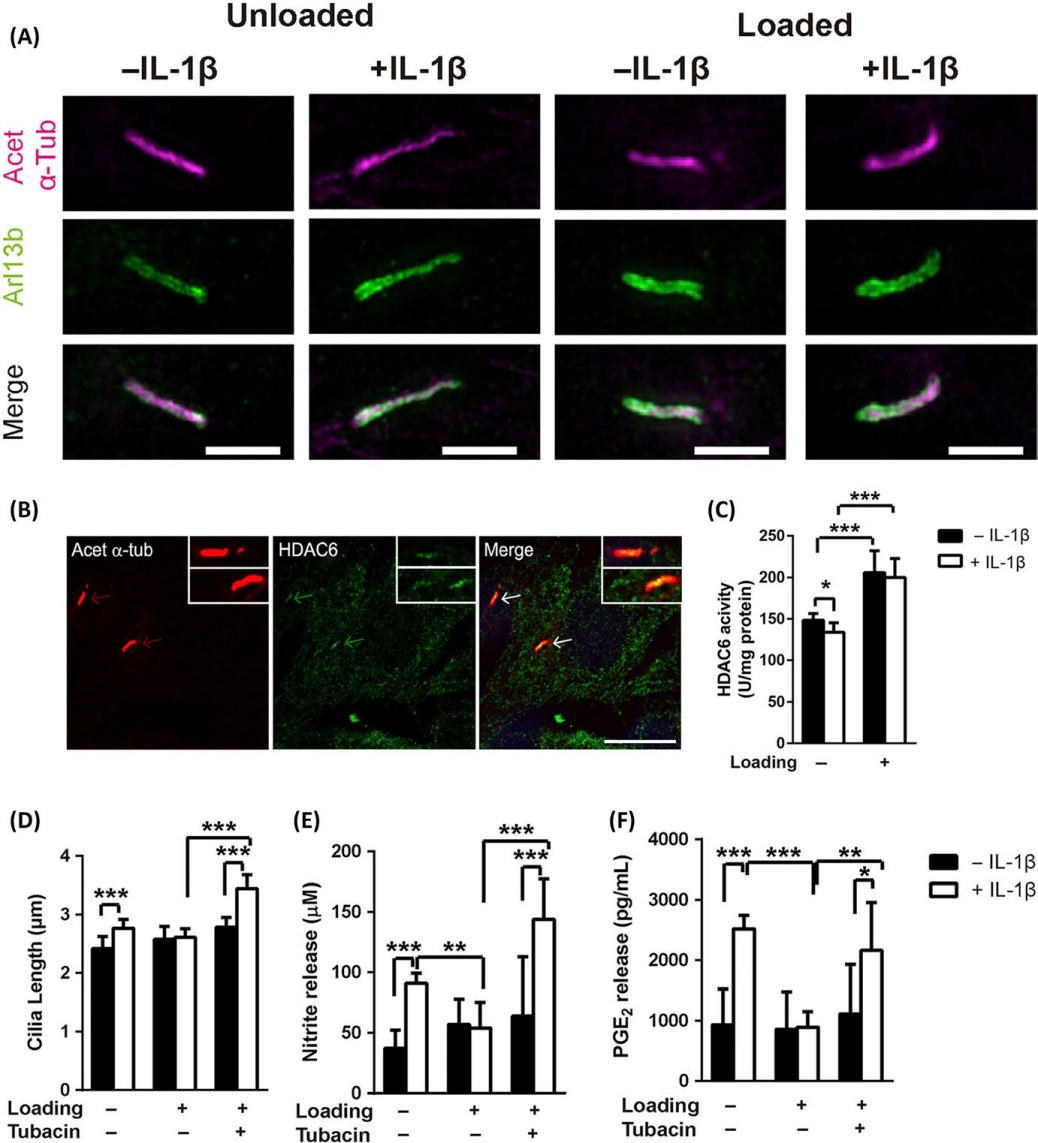
Mechanical stress inhibits cartilage inflammation via an HDAC6‐IFT‐dependent mechanism regulating primary cilia elongation. A, Images of chondrocyte primary cilia ± IL‐1β in or absence of mechanical loading (far‐red: acet‐α‐Tub and green: arl13b). B, Immunofluorescence for primary cilia (acet‐α‐Tub, red) and HDAC6 (green) co‐localization on the cilia axoneme in chondrocytes. C, HDAC6 activity for cells ± IL‐1β in or absence of mechanical loading. Statistical analyses of (D) cilia length, (E) nitrite release, and (F) PGE2 release. Reprinted with permission from Ref.^93^ Copyright 2019 Elsevier

Nowadays, the sensory functions of primary cilia in mechanical stress‐mediated signalling transduction have been widely recognized. However, there is no consensus on the regulatory mechanism of primary cilia‐mediated mechanical responses. A previous study reported that the expression of primary cilia in growth plate chondrocytes of proximal tibia could be upregulated after four days of 10% body weight loading in chicks. The results indicate that mechanical loading can enhance primary cilia expression in chondrocytes.^88^ The low‐frequency oscillatory mechanical stimulus can be produced by ultrasound, which can exhibit reversible changes in cells. In bovine articular chondrocytes, primary cilia elongation, axon bending and downstream ERK pathway activation can be observed under this ultrasound‐induced mechanical stress.^89^ The prevalence and length changes of primary cilia have also been proven to be related to the intensity and action time of mechanical stress.^38^ Primary cilia can be “stretched” by mechanical intervention, or “cut” by the excessive intensity of a mechanical stimulus. The variable regulation mode can reflect the capacity of cells to adapt to external mechanical stress.^90^, ^91^, ^92^ As Figure 3 shows, primary cilia are involved in the mechanical stress‐induced inhibitory regulation of chondrocyte inflammatory factor secretion in articular chondrocytes. The potential mechanism is the activation of HDAC6‐mediated microtubule acetylation and primary cilia elongation in an IFT‐dependent manner.^93^ Mechanical stress can also exert biological effects by regulating the interaction between primary cilia and autophagy in articular chondrocytes.^94^ Moreover, the loss of primary cilia in Col2aCre;ift88(fl/fl) mice can reduce the mechanical stiffness and compressive modulus of articular cartilage and eventually lead to the pathological changes of OA.^95^

Primary cilia are indispensable signal transduction units of the classical Hedgehog pathway, which is essential for regulating chondrocyte proliferation and differentiation.^96^ Mechanical stress can affect the activation of the Hedgehog pathway by acting on primary cilia. A previous study reported that hydrostatic stimulation could promote the activation of the Hedgehog pathway in chondrocytes, but it is blocked after disrupting the ciliary structure. The findings indicated that chondrocytes could respond to external mechanical stress by activating the primary cilia‐related Hedgehog pathway.^97^ Dicam, located at the primary cilia of chondrocytes, facilitates the proliferation and maturation of growth plate chondrocytes in limbs. The elongation of long bones can be regulated by Dicam through the promotion of primary cilia assembly and increase functional protein IFT88/Polaris expression, as well as enhancing Hedgehog/PTHrP signalling transduction.^98^

Additionally, the hyperactivity of the Hedgehog pathway has been found with tumorigenesis promotive effects, while primary cilia with normal functions can inhibit the excessive activation of Hedgehog signalling in neoplastic chondrocytes such as chondrosarcoma.^99^ The dysfunction of primary cilia can lead to the altered responsiveness of Hedgehog signalling which can contribute to the development of arthropathy, such as alkaptonuria.^100^, ^101^ These results highlight the importance of primary cilia in Hedgehog signalling transduction. Therefore, primary cilia are involved in mechanical stress‐mediated chondrogenesis and play essential roles in the regulation of cartilage development.

### 4.4 Primary cilia in cartilage tumorigenesis

Recently, the abnormal expression of primary cilia or ciliary dysfunction has been associated with the occurrence of neoplastic cartilage diseases. Some studies have reported that primary cilia disassembly can be observed in tumour‐like changes of cartilage, whether benign or malignant lesions. The abnormal activation of the Hedgehog pathway can lead to cartilage neoplasia, while primary cilia with normal structures and functions can inhibit cartilage tumorigenesis by regulating Hedgehog signalling transduction.^99^ In Ext1 gene mutant mice, the loss of primary cilia and cell cycle disorders can be observed, which causes cell polarity loss and promotes the transformation of benign osteochondroma to malignant chondrosarcoma.^20^ In chondrosarcoma, the expression of primary cilia is at a lower frequency than normal chondrocyte, and regulating the occurrence of primary cilia can affect the malignant biological properties of chondrosarcoma cells. The inhibition of intracellular HDAC6 activity can affect the acetylation modification of ciliary microtubules, which can regulate primary cilia assembly in chondrosarcomas through the Aurora A‐HDAC6 signal cascade and eventually downregulate the malignant properties of chondrosarcomas.^102^ Meanwhile, inhibition of the Hedgehog pathway can suppress the proliferation, migration and invasion capacities of malignant chondrosarcoma cells by disturbing ciliogenesis.^103^

In addition, some regulatory factors in cartilage development can achieve their functions by affecting primary cilia expression. It has been confirmed that fibroblast growth factor receptor 3 (FGFR3) mutations are closely related to chondrodysplasia. The aberrant activation of FGFR3 can disturb the elongation of primary cilia and affect the transport function of IFT20, while inhibiting FGFR3 can restore the normal length and function of primary cilia. The results reveal that the FGFR3 mutations inducing cartilage dysplasia have a close relationship with the dysfunction of primary cilia.^104^ PTH, another active growth factor, plays an important role in regulating cartilage development. Regulating PTH signalling can affect the malignant properties of chondrosarcoma by affecting primary cilia expression and function.^105^ These results may provide new ideas for exploring the pathogenetic mechanisms of cartilage tumorigenesis and the potential treatments for cartilage tumours.

### 4.5 Primary cilia in the cartilage disease osteoarthritis

Osteoarthritis is the most common degenerative disease of cartilage, which causes pain and the limitation of joint movements. Promoting cartilage repair and delaying cartilage degeneration possesses great value for osteoarthritis treatment. Recently, some studies have revealed that primary cilia play very important roles in regulating the pathogenesis of osteoarthritis.^106^, ^107^ Primary cilia disassembly and cartilage morphology changes can be found in many ciliary‐related gene mutants. For example, the abnormal development of cartilage can be found in the joints of Bardet‐Biedl syndrome mutant mice.^15^ The loss of primary cilia can also be detected in the cells of Col2aCre;Ift88fl/fl mutant mice, accompanied with osteoarthritis‐like cartilage changes. Furthermore, the expression of osteoarthritis marker proteins, such as COL‐X, RUNX2, MMP‐13 and ADAMTS‐5 increased, while a decrease in the hardness of cartilage tissue can be observed.^95^ Low‐level expression of GLI3 inhibitor in Col2aCre;Ift88fl/fl mutant mice might be associated with the abnormal activation of the Hedgehog pathway. The aberrant activation of the Hedgehog pathway can be initiated in mutant cartilage tissues.^108^ In Bbs^1M390R/M390R^ mutant ciliopathy mice, osteoarthritis‐like changes can be observed in the cartilage, and the dysfunction of primary cilia is involved in the regulation of degradative and pro‐inflammatory signalling transduction in these mutant mice.^109^ A previous study compared and analysed the differences in the prevalence and length of primary cilia between normal cartilage and osteoarthritis cartilage. The results revealed that the expression of primary cilia in the surface ciliated chondrocytes was lowest, and the length of cilia was the shortest in normal cartilage, while the prevalence and length of primary cilia in the deep layer chondrocytes increased correspondingly. In osteoarthritis cartilage, the incidence and length of primary cilia increased in the eroding articular from the surface to the tidal line with the severity of grading. These findings indicate that primary cilia are involved in the pathogenesis of osteoarthritis.^110^ Primary cilia can also be found in the regulation of chondrocyte inflammatory reactions.^111^ When chondrocytes were stimulated by IL‐1, primary cilia on the chondrocytes extended adaptively in a short time and controlled inflammatory factor release. After the knocking out of ciliated functional gene IFT88, the corresponding inflammatory response was suppressed remarkably.^36^

Primary cilia with a normal structure and function are beneficial for delaying the progression of osteoarthritis. Mechanical signalling transduction mediated by primary cilia can inhibit the expression of MMP‐1 and MMP‐13 in chondrocytes by transactivating CITED2 through the ATP‐purine calcium‐ERK1/2 signal axis, and MMP‐1 and MMP‐13 are considered the key proteins in regulating cartilage matrix catabolism, especially in the pathogenesis of osteoarthritis.^86^ Similarly, when chondrocytes are subjected to the appropriate intensity of mechanical stress, mechanical signals can be transduced by primary cilia and upregulate the activation of the Hedgehog pathway, as well as affect ADAMTS‐5 expression during cartilage development. However, the high intensity of mechanical stress can cause primary cilia dysfunction and lose the capacity of regulating Hedgehog signalling and ADAMTS‐5 expression in chondrocytes.^87^ Therefore, during the development of osteoarthritis, different intensities of mechanical stress can affect cartilage redifferentiation and development through primary cilia‐dependent regulatory signalling activation. Regulating the structure and function of primary cilia can be an effective choice for early intervention and delaying the progression of osteoarthritis. Additionally, a previous study reported that the injection of LiCl in an animal model of osteoarthritis could protect the joints and reduce cartilage damage by regulating primary cilia elongation and inhibiting the Hedgehog pathway in chondrocytes.^112^ Therefore, studies on primary cilia proteomics and cilia‐related regulatory factors can provide new choices for the treatment of osteoarthritis.

## 5 CONCLUSION AND PROSPECTS

Primary cilia, organelles with versatile functions, are involved in multiple regulatory mechanisms of cartilage development. The structural and functional integrity of primary cilia affects diverse life activities. For example, primary cilia disassembly and dysfunction can lead to achondroplasia, cartilage tumorigenesis and other degenerative diseases. Illustrating the regulatory mechanism of primary cilia in cartilage growth and development can provide new ideas for clarifying the pathogenesis of cartilage‐related diseases. However, there are still many problems to be resolved, for instance, whether the expression and function of primary cilia are related to cartilage matrix secretion; whether there is any quantitative relationship between the morphological changes of primary cilia and the mechanical forms; whether there is any dose‐effect relationship between the morphological changes of primary cilia and the mechanical resistant capacity of chondrocytes; what the specific roles of primary cilia in inflammatory responses are during the progression of osteoarthritis; and whether regulating the structure and function of primary cilia is useful to achieve the promotive effects on cartilage injury repair and regeneration in vivo. In summary, further research on the regulatory mechanism of primary cilia on cartilage growth and development will provide new ideas and prospects for the development of cartilage tissue engineering and new therapeutic choices for cartilage diseases.

## CONFLICTS OF INTEREST

The authors declare no conflicts of interest.

## AUTHORS CONTRIBUTION

Fenghua Tao and Ting Jiang wrote the manuscript and provided the critical revisions, Hai Tao and Hui Cao collected the update reference and drew the figures, Wei Xiang provided the conception and design of the study.

## Data Availability

Research data are not shared.
